# Supplementary material for: Genome-Wide Identification and Analysis of Stress Response of Trehalose-6-Phosphate Synthase and Trehalose-6-Phosphate Phosphatase Genes in Quinoa
Source: Int J Mol Sci. 2023 Apr 9;24(8):6950. doi: 10.3390/ijms24086950 (PMC10138372; doi:10.3390/ijms24086950)
Supplement: Supplementary file 1 [file ijms-24-06950-s001.zip › supplemental figures.pdf]

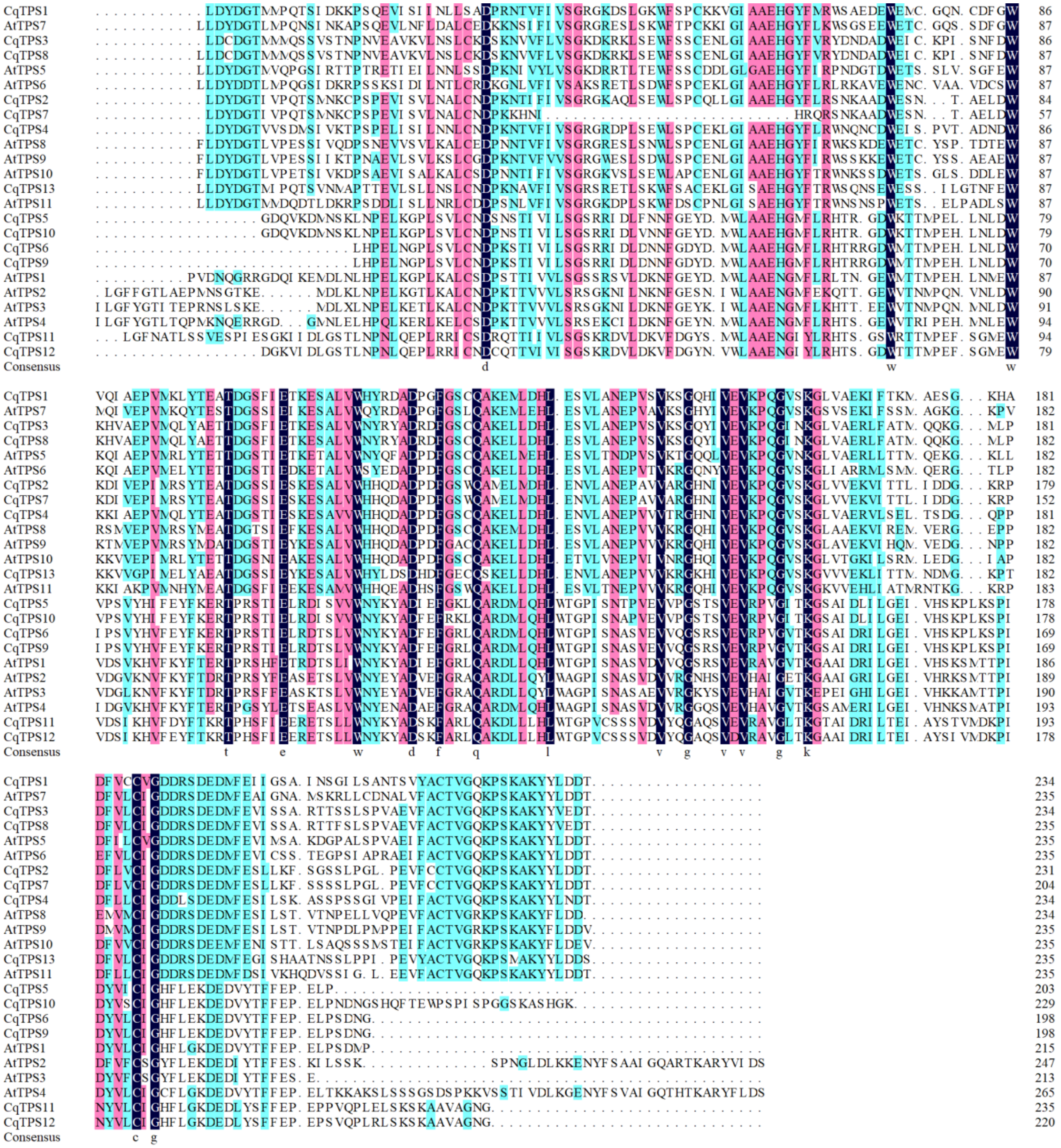

Supplemental Figure S1. Multi-sequence alignment of TPP domains in TPS proteins of *Arabidopsis* and quinoa. Different colors represent different homology levels. Black, identities =100%; pink, identities ≥ 75%; blue, identities ≥ 50%.

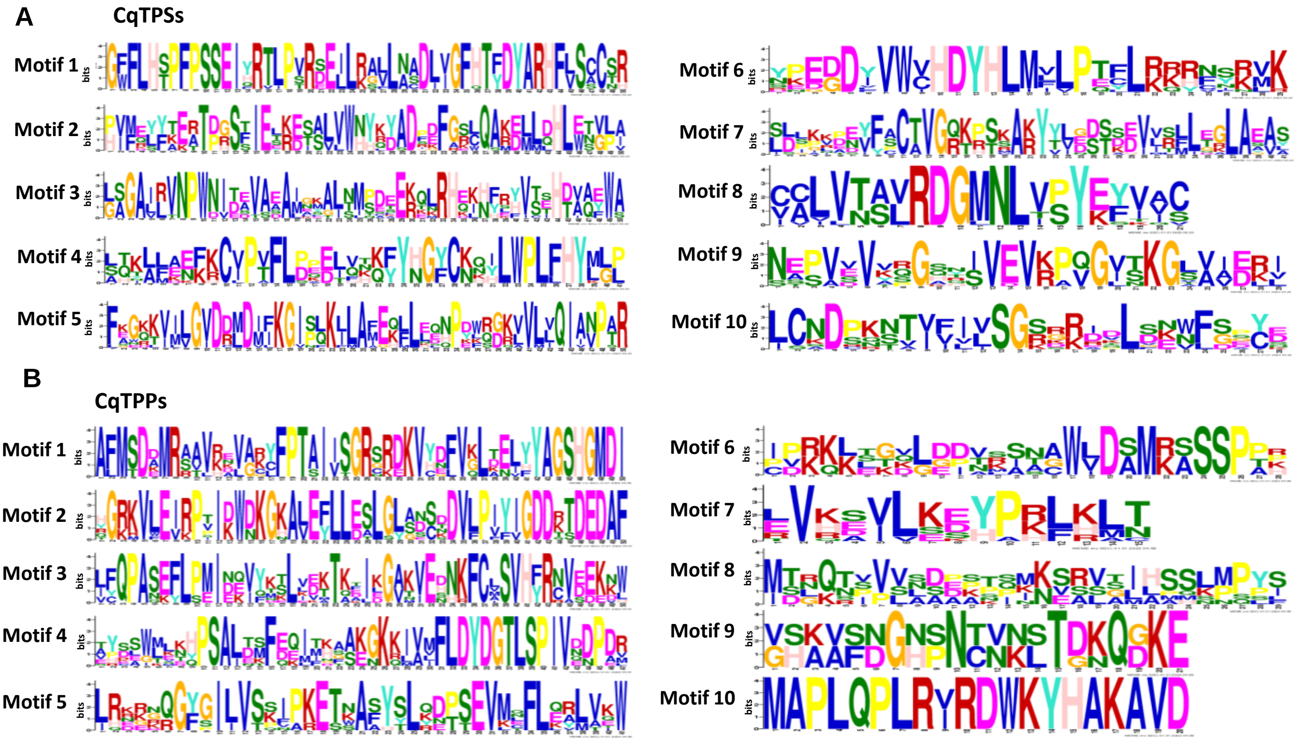

Supplemental Figure S2. Sequences of conservative motifs in CqTPS and CqTPP proteins. (**A**) Sequence analysis of CqTPS protein conserved motifs, color represents different amino acid residues, and larger font sizes represent more frequencies. (**B**)) Sequence analysis of CqTPP protein conserved motifs, color represents different amino acid residues, and larger font sizes represent more frequencies.

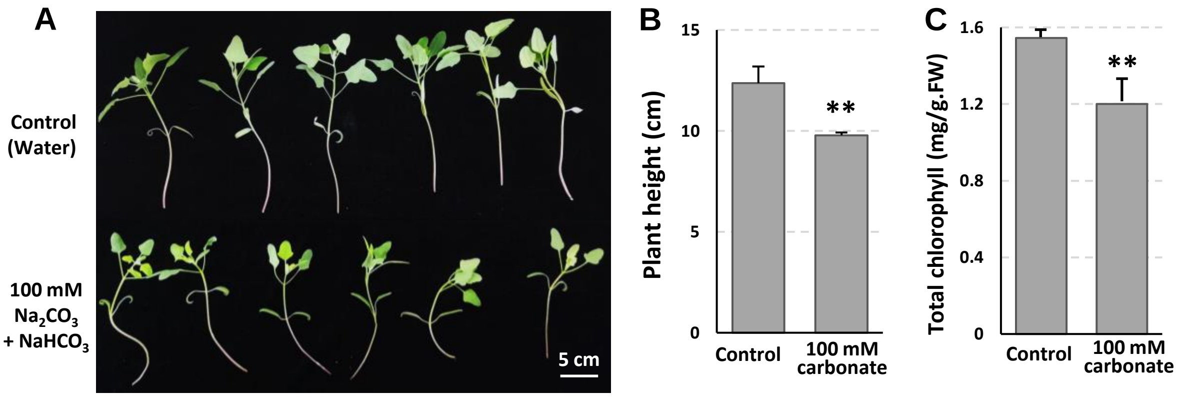

Supplemental Figure S3. Saline-alkali stress affected quinoa growth and development. (**A**) Quinoa plants undergone carbonate treatment. Two-week-old quinoa seedlings were treated with a solution containing 100 mM Na_2CO_3:$NaHCO_3$=1:9, once every 5 days, three times in total. (**B**) Statistics of plant height in A. (**C**) Statistics of total chlorophyll in quinoa leaves with or without carbonate treatment. **, $p < 0.01$.
