# Supplementary material for: Genome-Wide Identification and Analysis of Stress Response of Trehalose-6-Phosphate Synthase and Trehalose-6-Phosphate Phosphatase Genes in Quinoa
Source: Int J Mol Sci. 2023 Apr 9;24(8):6950. doi: 10.3390/ijms24086950 (PMC10138372; doi:10.3390/ijms24086950)
Supplement: Supplementary file 1 [file ijms-24-06950-s001.zip › supplemental table S5.pdf]

Supplemental Table S5. Primers used for RT-qPCR.

Gene ID	Forward (5'→3')	Reverse (5'→3')
AUR62020068 (UBQ9)	CAATGTCTACTACAACGAAGCG	CAGACTGACCAAACACAAAGTT
AUR62002838	ACTCAGATACGCAAAATTACGC	GACCAAGCTTATCTACGAGGAA
AUR62004026	AATGACACAAATGGAAAGGACG	GAGACTGTGGCTTGAGATAACT
AUR62004476	GGCGAAGTCTACTCAGAATGAT	GAAAATCTCGGGAAAACCTAGC
AUR62007199	GTATTGCAAGAATCAGACCGTC	CCATCTTCTCAGGCGTAAAATG
AUR62007314	TGGGGGTATTTAGATCTTGTCG	ATTGAGTAGGTGCGATATCGAG
AUR62008239	TGTAATTCCAAACGCAACCTTC	ACCGAGTTATTACGTGTTTTGC
AUR62012048	CAAGACAGAGCCTTTTAGGAGA	CAATTAGCATGGCTCTCAATCC
AUR62012679	TTTCTTTGTTGACCCAGTGATG	AAAGTAGAGAACACGCTACACA
AUR62012679	TTTCTTTGTTGACCCAGTGATG	AAAGTAGAGAACACGCTACACA
AUR62012986	TTAATGCCTTCCACCAAAAAGG	CTGCCATCTTTCTTATCAGCAC
AUR62012986	TTAATGCCTTCCACCAAAAAGG	CTGCCATCTTTCTTATCAGCAC
AUR62013843	GGGATGAACATGCTGTTACATC	TCAAACCGGAAATCTTGGAAAC
AUR62013843	GGGATGAACATGCTGTTACATC	TCAAACCGGAAATCTTGGAAAC
AUR62018691	CTATCATGGTGTCACGGTCTTA	GGAGCATCAGAGCTTACACTAT
AUR62020083	GGCATATGATGTTCCTATCCCA	GAAACAGATCAAAGTCCGTAGC
AUR62020390	GTGTAGATTTCATGGAATGGGC	CTTCGTCAATGGAAACCGTATC
AUR62021097	TGACATGAATGAAGCTTCGAAC	GTCTTGCTTATAATGGGTACGC
AUR62026544	GATTTTCCAAGAGATGTGACGG	GAAATTGCTGTGTTGTCGAGAT
AUR62028315	CTGCGTGCTTGTCAAAAATAAC	TCCACAATAAAGACAAATGCCG
AUR62029688	GCAGATGTAAAGCGAACGTTAT	GATGTAAACGCAAGGTATCCAC
AUR62034657	CAAAGCTATTGAAGGCATCTCC	GCTTGTGTTGGTAAATGGTCTT
