# Supplementary material for: Genome-Wide Identification and Analysis of Stress Response of Trehalose-6-Phosphate Synthase and Trehalose-6-Phosphate Phosphatase Genes in Quinoa
Source: Int J Mol Sci. 2023 Apr 9;24(8):6950. doi: 10.3390/ijms24086950 (PMC10138372; doi:10.3390/ijms24086950)
Supplement: Supplementary file 1 [file ijms-24-06950-s001.zip › supplemental tables S1 and S2.pdf]

Supplementary Table S1. Sequences of conservative motifs in CqTPS proteins.

Motif name	Sequence	Length (aa)
MOTIF1	GFFLHSPFPSSEIYRTLPVRDEJLRALLNADLVGFHTFDYARHFLSCCSR	50
MOTIF2	PVMEYYTERTDGSTIELKESALVWNYKYADPDFGSLQAKELLDHLETVLA	50
MOTIF3	LSGAIRVNPWNITEVAEAIGKALNMPDEEKQLRHZKHFRYVSEHDVAEWA	50
MOTIF4	LTKLLEEFKCVPVFLPPELVKKFYHGYCKQILWPLFHYMLP	41
MOTIF5	FKGKKVILGVDDMDIFKGISLKJLAFEKLLEZNPDWRGKVVLVQIANPAR	50
MOTIF6	YPEDDYVWVHDYHLMVLPTFLRKRNSRVK	29
MOTIF7	LDLKKPEVFACTVGQKPSKAKYYLEDSSEVVSLLEGLAEAS	41
MOTIF8	CALVTAVRDGMNLVPYEYVAC	21
MOTIF9	NEPVEVVRGSNIVEVKPQGVTKGLVIERI	29
MOTIF10	LCNDPKNTIFIVSGSRRDDLSEWFSPYE	28

Supplementary Table S2. Sequences of conservative motifs in CqTPP proteins.

Motif name	Sequence	Length (aa)
MOTIF1	AFMSDDMRAAVREVAKYFPTAIISGRSRDKVYDFVKLTELYYAGSHGMDI	50
MOTIF2	HGRKVLEIRPTIDWDKGKALEFLLESLGLANSDDVLPIYIGDDKTDEDAF	50
MOTIF3	JFQPASEFLPMIDEVYKSLVEKTKEIKGAKVEDNKFCLSVHFRNVEEKNW	50
MOTIF4	AYSSWLLKHPSALDSFEZITKAAKGKKIVMFLDYDGTLSPIVDDPDR	41
MOTIF5	LRKRNQGYGILVSKIPKETNASYSLQDPSEVMEFLZRLVKW	50
MOTIF6	IPKKKTGKLDDVKSNAWLDAMKASSPPK	29
MOTIF7	EVKEVLKEYPRLKLT	41
MOTIF8	MTRQTVVVADEPTPMKSRVAIHSSLPPYS	21
MOTIF9	GHKVFDGNPNCNKLTDKQGKE	29
MOTIF10	MAPLQPLRIRDWKYHAKAVD	28
